# Therapeutic Targeting of TLR4 for Inflammation, Infection, and Cancer: A Perspective for Disaccharide Lipid A Mimetics

**DOI:** 10.3390/ph16010023

**Published:** 2022-12-23

**Authors:** Holger Heine, Alla Zamyatina

**Affiliations:** 1 Research Group Innate Immunity, Research Center Borstel—Leibniz Lung Center, Airway Research Center North (ARCN), German Center for Lung Research (DZL), Parkallee 22, 23845 Borstel, Germany; 2 Department of Chemistry, University of Natural Resources and Life Sciences, Muthgasse 18, 1190 Vienna, Austria

**Keywords:** immunomodulation, carbohydrates, lipopolysaccharide, lipid A, adjuvant, TLR4 antagonist, TLR4 agonist, innate immunity

## Abstract

The Toll-like receptor 4 (TLR4) signaling pathway plays a central role in the prompt defense against infectious challenge and provides immediate response to Gram-negative bacterial infection. The TLR4/MD-2 complex can sense and respond to various pathogen-associated molecular patterns (PAMPs) with bacterial lipopolysaccharide (LPS) being the most potent and the most frequently occurring activator of the TLR4-mediated inflammation. TLR4 is believed to be both a friend and foe since improperly regulated TLR4 signaling can result in the overactivation of immune responses leading to sepsis, acute lung injury, or pathologic chronic inflammation involved in cancer and autoimmune disease. TLR4 is also considered a legitimate target for vaccine adjuvant development since its activation can boost the adaptive immune responses. The dual action of the TLR4 complex justifies the efforts in the development of both TLR4 antagonists as antisepsis drug candidates or remedies for chronic inflammatory diseases and TLR4 agonists as vaccine adjuvants or immunotherapeutics. In this review, we provide a brief overview of the biochemical evidences for possible pharmacologic applications of TLR4 ligands as therapeutics and report our systematic studies on the design, synthesis, and immunobiological evaluation of carbohydrate-based TLR4 antagonists with nanomolar affinity for MD-2 as well as disaccharide-based TLR4 agonists with picomolar affinity for the TLR4/MD-2 complex.

## 1. Introduction

**Figure 1:**
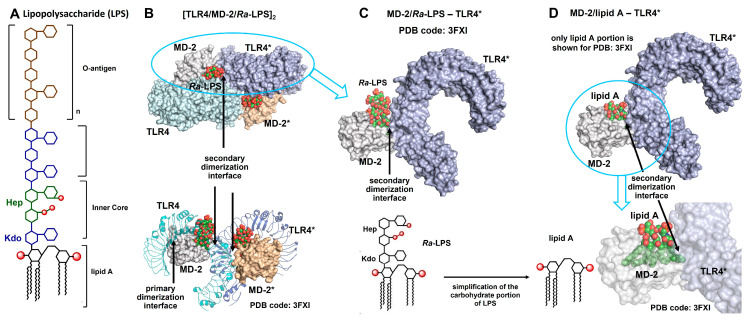
LPS-induced TLR4 complex dimerization. (**A**) Schematic representation of LPS; (**B**) crystal structure of TLR4/MD-2/LPS complex, PDB code: 3FXI; (**C**) formation of a secondary dimerization interface between TLR4/MD-2/LPS and the second TLR4* complex; (**D**) positioning of *E.coli* lipid A in the binding pocket of MD-2 and lipid A participation in the dimerization process. Images were generated with PyMol.

When Pfeiffer in 1892 coined the term “Endotoxin” for a toxin associated with the body of the bacteria and exhibiting the deadly effects of heat-killed cholera bacteria, he could not possibly have known how peculiar and fascinating this molecule would turn out to be [1]. It took more than 50 years to identify this “Endotoxin” as the lipopolysaccharide (LPS) of Gram-negative bacteria [2]. LPS is a bacterial glycan which is composed of three major motifs: a membrane-anchored lipid A, a Kdo- and heptose-containing core oligosaccharide, and a variable O-antigen (Figure 1A). The lipid A portion represents the “endotoxic principle” of LPS [3,4,5,6], which was already assumed by Westphal and Lüderitz (1954) and finally proven by Galanos et al. through the generation and subsequent functional analysis of synthetic lipid A structures [7]. The structure of a canonical endotoxic lipid A of *E. coli* relies on a β(1→6)-linked D-glucosamine disaccharide that is hexa-acylated with two long-chain (*R*)-3-alkanoyloxyalkanoyl lipid residues attached at the “distal” GlcN ring and two (*R*)-3-hydroxyalkanoic fatty acids linked to the “proximal” GlcN moiety and carrying two phosphate groups [8,9,10]. The lipid chains of pro-inflammatory active lipid A variants normally have 12 to 14 carbon atoms. Generally, the endotoxic activity of lipid A is dictated by the following parameters: (i) the chemical structure, length, number, and distribution pattern of lipid chains bound to the disaccharide backbone and (ii) the phosphorylation status of each sugar unit [11].

In 1999, the cell surface receptor for LPS had finally been discovered as Toll-like receptor 4 [12,13]. However, the signaling induced by LPS through TLR4 proved to be more complex than with all other TLRs. A sequential event leads to the extraction of LPS molecules by the serum constituent LPS-binding protein (LBP), the transfer to the membrane-tethered GPI-anchored receptor CD14, and the binding to a TLR4 co-receptor, myeloid differentiation 2 (MD-2) [14,15]. MD-2 is a small, secreted molecule that binds to and becomes physically associated with TLR4 [16]. In turn, LPS binding to MD-2 leads to the assembly of a ligand–receptor homodimer formed of two copies of the TLR4/MD-2/LPS complex (Figure 1B) [17,18,19]. Upon generation of [TLR4/MD-2/LPS]_2_ homodimers, the intracellular signaling is initiated through conformational changes in the Toll/IL-1R (TIR) domain of TLR4, enabling the subsequent attachment of signal transduction adapter molecules. Among the TLRs, TLR4 has a special role, as it is the only TLR that uses both signaling adapter molecules, MyD88 (myeloid differentiation primary response 88) [20,21]) and TRIF (TIR (Toll IL-1R)-domain-containing adaptor-inducing interferon-β) [22]. This exclusivity allows TLR4 to drive two different time-shifted sets of immune responses: The first set, starting at the plasma membrane, is MyD88-dependent and leads to a rapid induction of pro-inflammatory cytokines. The second set, however, requires internalization, is TRIF-dependent, and signals from endosomal membranes for the induction of a type I interferon response.

Although the TLR4 complex is a major sensor of Gram-negative bacterial LPS, it can also be activated by various endogenous danger-associated molecules (DAMPs) [23,24] released from lytic cells during host tissue injury or viral infection. Indeed, it has been shown over the last decade that self-encoded molecules derived from damaged or necrotic tissues such as oxidized phospholipids [25], high-mobility group box 1 protein (HMGB1) [26], hyaluronic acid fragments [27], or fibronectins [28] are also able to signal through activation of TLR4. To make LPS-induced cell activation even more complex, it was revealed in 2014 that TLR4 is not the only LPS receptor but that a specific TLR4-independent cytosolic recognition of Gram-negative bacteria and their LPS exists: intracellular caspases (caspase-11 in mouse and caspase-4/5 in humans) were demonstrated to bind LPS and trigger a non-canonical inflammasome activation [29,30,31,32]. Inflammasome activation leads to pyroptosis and to the production and release of IL-1β, IL-18, and IL-37—cytokines which are heavily involved in inflammation and inflammatory disorders [33,34].

## 2. Species-Specific Activity of LPS

Another peculiarity of LPS biology that is not seen to this extent with any other pathogen-associated molecular pattern (PAMP) is its species-specific activity. Much of the in vivo functional analysis of LPS-induced signaling, gene expression, and cytokine production in monocytes and macrophages has been performed using different mouse models. Although the general LPS receptor complex is conserved between humans and mice (and mammals in general), there are some striking differences that impact ligand recognition [35,36,37].

For example, the tetra-acylated biosynthetic precursor of *E. coli* lipid A, also known as lipid IVa (and its synthetic analogue, compound 406), has endotoxic activity in mice in vitro as well as in vivo [38] but is completely inactive in human cells [39,40,41]. However, despite being inactive at human (h-)TLR4, the binding to hTLR4 still takes place and therefore this structure has been identified as a very good antagonist in the human system [42]. Similarly, the *N. meningitidis* LpxL1 LPS mutant carrying penta-acylated lipid A activates the murine TLR4/MD-2 complex equally efficiently as a wild-type hexa-acylated LPS, whereas it elicits no or a significantly reduced TLR4-mediated response in native human immune cells and can even act as an antagonist at hTLR4 [43]. The species-specific activity of LPS goes even beyond man and mice: the fivefold-acylated *R. sphaeroides* lipid A is a potent antagonist in human and mouse cells but activates cells of hamster [44] and equine origin [45,46].

These findings started a new research field of the identification and synthetic construction of stable and adjustable LPS/lipid A antagonists in order to explore their therapeutic potential.

## 3. Therapeutic Potential of TLR4 Antagonists

The first disorder where LPS antagonists come to mind is **sepsis**. In 2017, the estimated number of sepsis-related deaths worldwide was 11 million [47]. Despite a decline in overall incidence, it is still a major public health burden associated in many cases with primary or secondary infections by Gram-negative bacteria and thus their LPS as a virulence factor. Based on promising in vivo data in animal studies and a phase 2 trial, a synthetic antisepsis drug candidate E5564 (Eritoran), an LPS antagonist based on the structure of *R. sphaeroides* lipid A, was tested in a phase 3 trial but failed to improve 28-day mortality [48]. Among the reasons for this failure are significant differences in TLR4 and caspase 4/5/11 signaling and expression in humans and mice. At the time Eritoran was tested in septic patients, the involvement and the importance of caspases in LPS-triggered inflammation was not yet known.

However, the above-mentioned failure of Eritoran in sepsis trials was not the end of its possible use. **Flu pandemics** are another world-wide public health threat with high morbidity and mortality [49]. Although viruses are detected by other PRRs than TLR4 (TLR3, 7, 8, RIG-I), in the more severe outcomes of viral infection such as acute lung injury (ALI), TLR4 signaling is crucially involved through the generation of oxidized phospholipids (OxPAPC) which can also activate TLR4 [25]. It has been proven that administration of Eritoran protects mice from lethal influenza infection by blocking OxPAPC-dependent activation of TLR4 and that the harmful TLR4-mediated signaling can be inhibited also in this case by TLR4 antagonists directly interacting with MD-2 [50].

Another chromatin-associated protein-derived DAMP, HMGB1, which can be secreted from dying cells during **viral infection**, is also sensed by the TLR4 co-receptor MD-2 [51]. The viral-infection-mediated release of HMGB1 triggers pro-inflammatory responses similar to those of LPS and thus supports the significance of the therapeutic potential of TLR4/MD-2 antagonists for symptom release in viral disease. It is assumed that the TLR4 axis is also involved in the detrimental effects of respiratory syncytial virus (RSV) [52], West Nile virus [53], filovirus [54], and influenza virus infections [55,56].

Morbidity and mortality in the course of **acute viral disease** is frequently associated with excessive inflammation triggered by the release of elevated levels of pro-inflammatory cytokines and chemokines which can lead to life-threatening complications such as acute respiratory distress syndrome or ALI, circulatory shock, and myocarditis. Moreover, an upregulation of the TLR4-mediated pro-inflammatory signaling plays an important role in the pathogenesis of **SARS-CoV-2** [57,58,59]. The innate immune responses to a viral-infection-induced cytokine storm are often systemic and result in sepsis syndrome mimicking the symptoms of Gram-negative bacterial sepsis as reported for SARS-CoV-2 disease [60,61]. Indeed, TLR4 seems to be a crucial contributor to the pathology and severity of COVID19 [62,63,64], especially in patients with preexisting cardiovascular comorbidities [65] which can be associated with preceding or inducible endotoxemia [66] and/or the ability of SARS-CoV-2 spike protein to bind and transport bacterial LPS [62].

LPS is a very potent driver of inflammation [67] and thus is clearly involved in many chronic inflammatory disorders such as **atherosclerosis** [68,69,70,71]. Here, in particular, the capability of LPS to engage both TLR4 and inflammatory caspases is of crucial importance. A main source of LPS is the microbiome, and dysbiosis leads to impairment of the gut barrier enabling LPS translocation in the blood stream [68]. A number of studies address the role of TLR4 in the regulation of skin defence mechanisms during cutaneous tumorigenic inflammation [72]. Pharmacologic inhibition of the TLR4-driven responses enhanced the skin’s photoprotective capacity, attenuating cutaneous stress signaling, and suppressed UV-induced skin tumorigenesis in mice [73].

In rheumatoid **arthritis** patients, the endogenous TLR4 agonist HMGB1 was detected in increased levels in the serum and synovial tissues, providing another link to TLR4 signaling in inflammatory disorders [74]. Similarly, other DAMPs were shown to propel inflammation through the activation of TLR4 in rheumatoid and osteoarthritis, which highlights the inhibition of TLR4-mediated signaling as a potential therapeutic option [75,76,77].

The contribution of LPS and TLR4 signaling to disease pathology is often not clearly determined and can be positively or negatively associated with a disease as in the case of COPD (chronic obstructive pulmonary disease) [78] and pulmonary fibrosis [79]. In COPD, the expression of TLR4 is elevated, and TLR4, among other TLRs, is also involved in cigarette-smoke-induced inflammation [80]. Polymorphisms that reduce the functionality of TLR4 have been shown to either contribute to COPD development (TLR4-T399I) [81] or reduce COPD severity (TLR4-D299G) [82].

TLR4-mediated production of pro-inflammatory cytokines is also involved in different types of **myocardial inflammation**, and a number of trials evaluated TLR4 as a potential target for improvement of disease outcome and severity [83,84]. Inhibition of TLR4-driven inflammation, which is a major mortality factor in trauma–hemorrhage patients, showed positive results in overcoming hemorrhage-induced cardiac dysfunction in vivo [85].

Epidemiologic studies clearly indicate that early contact with microbes and their LPS decrease the occurrence of **asthma and allergies** in later life [86,87], corroborating the “hygiene hypothesis” introduced by Strachan [88]. However, the role of LPS itself in asthma and allergy development is not that clear because, depending on the timing and the amount of LPS exposure, it can also increase their development [89,90]. To add to this complexity, certain allergens are directly involved in TLR4 signaling: Der p 2, the main allergen of the house dust mite, has not only structural but also functional homology to MD-2 and thus can replace MD-2 in the LPS/MD-2/TLR4 receptor complex [91]. This self-adjuvant effect can play a major role for the allergenicity of other members of the MD-2-like lipid-binding proteins as well [92]. TLR4 was confirmed as a therapeutic target in the management of airway remodeling in house dust mite-induced chronic asthma. Inhibition of TLR4 signaling showed positive effects in a murine chronic asthma model through hampering of the Th2 response and suppression of airway remodeling [93]. In general, the TLR4 signaling pathway was suggested to protect mice against the development of allergic asthma [94]. In contrast to house dust mite allergen, the cat dander protein Fel d 1 enhances TLR4 signaling not by mimicking the TLR4 co-receptor MD-2 or binding to the TLR4/MD2 complex but instead efficiently binding LPS and, therefore, contributing to a lipid transfer mechanism of LPS to MD-2/TLR4 [95]. Similar properties are suggested for a lipocalin-class protein, the dog allergen Can f 6, which can enhance innate immune signaling by LPS and foster airway hypersensitivity in asthma [95].

## 4. Therapeutic Potential of TLR4 Agonists

In addition to the role of TLR4 in the diverse pathologies mentioned above, there is an increasing number of publications indicating the involvement of LPS and/or TLR4 signaling in **Alzheimer’s** disease [96,97,98]. TLR4 agonists were suggested to have a therapeutic potential for Alzheimer’s disease by stimulating the immune system to clear amyloid β. Systemic administration of a weak TLR4 agonist monophosphoryl lipid A (MPLA) led to a substantial reduction in amyloid β load in the brain and to a significant improvement in Alzheimer’s-disease-related pathology in mice [99]. Likewise, repeated injections of LPS to transgenic mice to provoke mild but permanent stimulation of the TLR4 axis without inducing pathogenic neuroinflammation led to augmentation of neuronal autophagy, improved cognitive function, and prompted attenuation of the Alzheimer’s-disease-related symptoms [100].

Though vaccine **adjuvants** have long been used to either compensate for poor antigen immunogenicity or to reduce the amount of an antigen required for protection, very few have been licensed for human use. The most promising adjuvants that can promote improved innate and adaptive immune responses are those with a preferential Th1 bias, including agonists of certain PRRs and TLR4 in particular [101,102]. Recombinant subunit vaccines supplemented with various formulations (in combination with aluminum salts (AS04) or squalene (AS03)) of the TLR4 agonist MPLA [103] were proven to be more effective than the live attenuated vaccines, thus demonstrating a central role of adjuvants in vaccine development. Currently, MPL^®^ has been approved for use in several human viral vaccines [104]; however, substantial batch-to-batch variations in the content of vaccine-grade MPL^®^ adjuvant (obtained by acid and base hydrolysis of the LPS of *S. enterica* serovar Minnesota Re595) and contaminations with underacylated TLR4-antagonizing lipid A variants bring about serious limitations [105]. It is well known that a moderate activation of TLR4 by exogeneous PAMPs comprises an important mechanism of defense against viral infection in an immunocompetent host by warranting the induction of specific pro-inflammatory signaling pathways [56,106,107]. Therefore, adjuvants targeting TLR4 might provide additional advantages by mounting an intrinsic protective response aimed at the induction of immunological memory. Thus, there is a huge demand for the discovery of more efficient TLR4 agonists other than LPS as non-toxic amplifiers of vaccine efficacy.

It has been proposed that TLR4-driven inflammation could play a dual role in **cancer**: whereas chronic inflammation (e.g., by low doses of LPS released in chronic infection or by endogenous DAMPs produced during infection or sterile inflammation) can facilitate tumor growth and progression [108,109,110,111,112,113,114], induced activation of the TLR4 axis improves antitumor responses and is associated with a positive prognosis, suppression of tumor growth, and reduction in cancer metastasis [115,116,117,118,119]. Based on these results, induction and/or amplification of the TLR4 signaling pathway was suggested as a potential treatment strategy in specific cancers [116,120,121,122,123,124].

Indeed, it has been known as early as the 13th century that acute inflammation has the potential to inhibit cancer progression or even eradicate solid tumors: this hypothesis was supported by reports on spontaneous cancer regression following acute bacterial infection [125]. In the early 20th century, a bone surgeon, William B. Coley, demonstrated significant success against advanced-stage bone and soft-tissue sarcoma by applying mixtures of live or killed bacteria to induce topical and systemic acute inflammation [126,127]. The molecular mechanism of this curative effect was not addressed in those times, and the treatments with the preparations of whole bacteria were strongly toxic and often lethal. However, Coley’s treatment had a higher success rate (57% complete tumor remission) than contemporary chemo- and radiotherapy [128]. According to current knowledge, the anticancer effect of “Coley vaccine” was related to the activation of TLR2 and TLR4 signaling by its two components, the Gram-positive *S. pyogenes* and Gram-negative *S. marcescens*, respectively.

TLR4 is widely expressed in tumor cells as well as the cells of the tumor microenvironment such as tumor-associated macrophages (TAMs), cytotoxic T cells, myeloid-derived suppressor cells, and natural killer cells. Growing evidences demonstrate that the activation of TLRs in the tumor microenvironment can break the tumor-induced immune tolerance and boost antitumor immunity [129,130]. Furthermore, augmented expression of TLR4 in several immunogenic cancer types predicts positive survival prognosis [131]. In particular, switching TAMs from a pro-tumorigenic and immunosuppressive (M2/repair-type) to an anti-tumorigenic and immunostimulatory (M1/killer-type) phenotype using appropriate TLR agonists is an emerging strategy towards immunity-based approaches for the management of solid cancers. Immunostimulatory M1-type macrophages can stimulate effector cells such as Th1-type cytotoxic T cells and promote tumor regression [132,133,134,135,136].

In fact, TLR4 is increasingly considered a promising therapeutic target for solid cancers, and TLR4 activation by low doses of endotoxic LPS from *E.coli* showed therapeutic promise in inhibiting the progression of osteosarcoma [115], lymphoma [137], subcutaneous glioma [138], and osteosarcoma [115]. TLR4 activation in the microenvironmental cells mediated anticancer effects by stimulating CD8-positive cytotoxic lymphocytes [115] and altering the maturation state of tumor-surrounding dendritic cells [122]. Moreover, preparations of a weak TLR4 agonist MPLA (or its synthetic analogue GLA) restored the intratumoral activity of cytotoxic T cells and elevated pulmonary NK cell cytotoxicity, which led to tumor eradication and a reduction in cancer metastasis [119,139]. Nevertheless, preclinical development of LPS-based cancer treatment turned out to be challenging due to possible adverse effects or clinical inactivity as a result of LPS heterogeneity [120].

Therefore, the development of molecularly defined, homogeneous TLR4 agonists which are safe and well tolerated in humans as potential stand-alone therapeutics for cancer or as supplements in checkpoint inhibitor treatment is highly required. For other tumor types, such as breast or colon cancer, where the overexpression of TLR4 is frequently associated with a negative prognosis, the development of TLR4-inhibiting strategies targeting its co-receptor protein MD-2 could hold therapeutic promise [110,113,140,141].

**Figure 2:**
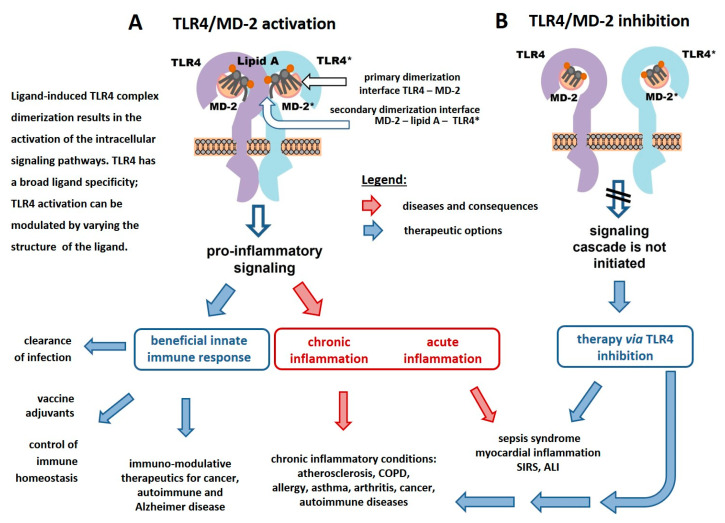
Schematic representation of lipid A-induced activation (**A**) and inhibition (**B**) of the TLR4/MD-2 complex and the options for therapeutic modulation of TLR4-mediated signaling.

However, the exact molecular mechanisms driving these different pathologies and the varying importance of LPS- and DAMP-dependent TLR4- or caspase-4/5-induced signaling and cellular activation are often not clear at all. Thus, the design, chemical synthesis, and functional studies of molecularly defined carbohydrate-based LPS mimetics that are capable of either interfering with the TLR4 signaling or enhancing and modulating the TLR4-driven pro-inflammatory responses provide a versatile tool for studying the underlying mechanisms of TLR4-mediated inflammation and has a great therapeutic potential (Figure 2).

## 5. Challenges in Development of Synthetic TLR4 Ligands

The chemical complexity of LPS and its truncated mutants (*Ra-* and *Re*-LPS) [142], representing the minimal structure needed for full pro-inflammatory activity and proper recognition by the TLR4 complex, renders the synthesis of possible drug candidates a formidable challenge. The complexity and poor predictability of LPS-TLR4 structure–activity relationships [143] as well as evidences for enormous influence of subtle variations in the chemical composition of lipid A (e.g., minor variations in the length of lipid chains) on the expression of TLR4-mediated activity makes rational prediction of biological properties of lipid-A/LPS-derived drugs hardly possible. In addition, the inherent conformational plasticity of LPS-recognizing protein MD-2, which markedly changes its conformation contingent on the structure of a bound ligand [144], makes classical “rational drug design” inefficient in this case. Finally, the species specificity in recognition of the diverse ligands by the TLR4 complex hampers straightforward extrapolation of data obtained in vitro and in animal models for clinical trials. Along these lines, pharmacological exploitation of the LPS/TLR4 complex and rational design of drugs targeting the co-receptor protein MD-2 is far away from being straightforward.

## 6. Co-crystal-Structure-Based Design of Disaccharide Lipid A Mimetics (DLAMs)

**Figure 3:**
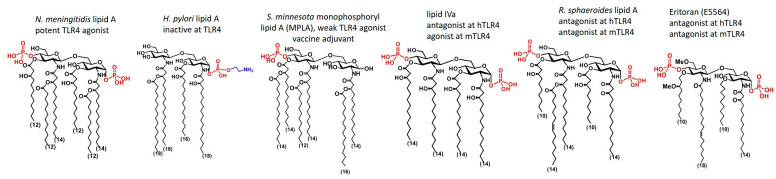
Chemical structure of several lipid A variants.

The chemical structure of lipid A is highly conserved within species and relies on a β(1→6) disaccharide backbone (GlcN-β(1→6)-GlcN) that is multiply acylated by optically active β-hydroxy fatty acids at equatorially oriented OH groups (positions 2,2´ and 3,3´) (Figure 3). The positions 4 and 6´ in lipid A are free (whereas position 6´ is reserved for linking the core sugars) and the residual OH groups at positions 4´ and 1 are commonly phosphorylated. The secondary 4´-linked phosphate group at the distal GlcN ring is absent in several lipid A modifications which significantly downregulates the TLR4-mediated signaling [9,10]. Similarly, hydrolysis of the intrinsically labile anomeric 1-phosphate group at the proximal GlcN ring leads to attenuation of endotoxic activity which can be applied in biotechnological manipulations of LPS/lipid A structures with the aim to reduce possible adverse effects as in the case of MPLA. The hexa-acylated lipid A of *N. meningitidis* and *E. coli* is the most potent TLR4 agonist known to date, whereas a low degree of acylation is characteristic for lipid A variants with either TLR4 antagonistic properties (as in the case of the tetra-acylated lipid IVa [42] and penta-acylated nonpathogenic lipid A of *R. sphaeroides* [145]) or no TLR4-mediated activity (Figure 3).

**Figure 4:**
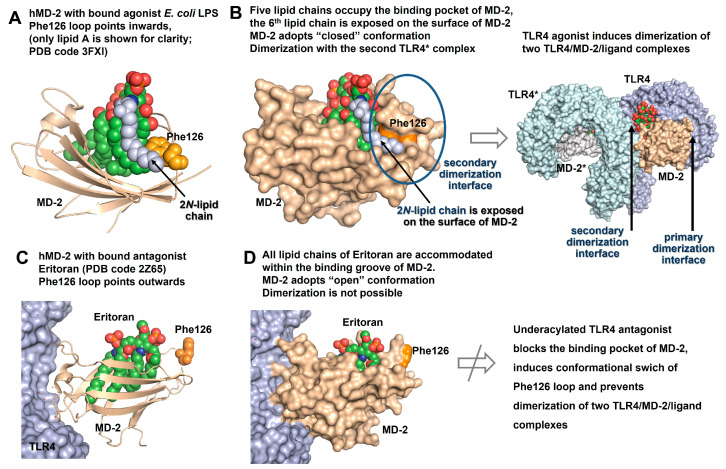
Comparison of co-crystal structures of TLR4/MD-2-bound agonist and antagonist LPS/lipid A variants. (**A**) Co-crystal structure of hTLR4/MD-2/*Ra*-LPS (*E. coli*) complex (PDB code: 3FXI, only hMD-2 with bound lipid A portion of LPS is shown for clarity). The exterior positioning of the 2*N*-acyl chain (colored blue) of hTLR4/MD-2-bound lipid A is supported by interaction with Phe126 (colored orange) which points inwards due to ligand-driven conformational rearrangement in hMD-2. (**B**) Five lipid chains of hexa-acylated lipid A are accommodated in the binding pocket of hMD-2. The 6th 2*N*-linked hydroxyacyl residue is excluded from the binding pocket and, together with a side chain of Phe126, contributes to the formation of a secondary dimerization interface. The LPS binding induces dimerization of two TLR4/MD-2/LPS complexes. (**C**) All lipid chains of a tetra-lipidated TLR4 antagonist Eritoran are intercalated into the binding groove of hMD-2 (PDB code: 2Z65). Phe126 (colored orange) is exposed to solvent. (**D**) hMD-2 with bound antagonist ligand adopts an “open” conformation that results in prevention of dimerization. Images were generated with PyMol.

In line with the published co-crystal structures of *E.coli Ra/Re*-LPS bound to h- or m-TLR4/MD-2 complexes (PDB codes: 3FXI, 3VQ1), respectively, five out of six lipid chains of a typical hexa-acylated lipid A are spaciously incorporated into the hydrophobic binding cavity of the co-receptor protein MD-2 (Figure 4A). The sixth lipid chain (according to the X-ray structure, the 2*N*-linked 3-hydroxyalkanoyl chain attached at the proximal GlcN moiety) is displayed on the surface of MD-2 as it does not fit into the tightly occupied binding pocket [17,146]. This creates a so-called “heterodimerization” or “secondary dimerization interface” which involves a hydrophobic patch on the surface of MD-2 (Phe126, Y131, Leu-rich repeats), an exposed 2*N*-hydroxyalkanoyl chain of lipid A and the Leu-rich repeats (incl. Phe440, Phe463 for hTLR4 and Phe438, Phe461 for mTLR4) of the second TLR4* [17,147]. The formation of a hexameric [TLR4/MD-2/LPS]_2_ complex is preceded by significant ligand-driven conformational changes in MD-2 which force the protein in its “closed” conformation able to undergo dimerization (Figure 4B) [144,148]. The phosphate groups of lipid A (1- and 4′-linked) are crucial for binding to hMD-2 where they interact with several positively charged side chains on the rim of the binding groove (mostly Lys and Arg) and are also important for establishing a secondary dimerization interface with the second TLR4* complex [149]. The absence of one of the phosphate groups results in a 100-fold drop in biological activity, partially due to a lack of efficient dimerization, and is manifested by reduced cytokine release [150,151].

The situation is quite different for protein-bound underacylated lipid A variants such as lipid IVa and Eritoran (Figure 3) where all lipid chains are fully incorporated into the binding cleft of hMD-2 (Figure 3C) [42,152]. This type of binding brings hMD-2 into an “open” conformation with the Phe126 loop exposed to solvent which makes dimerization with the second hTLR4* complex impossible and thus renders these lipid A variants potent hTLR4 antagonists (Figure 4D). Accordingly, TLR4 antagonists can tightly bind to the hydrophobic pocket of MD-2 without initiating pro-inflammatory signaling and compete with the natural ligand LPS in occupying the same site on the co-receptor protein.

The chemotype-specific TLR4 activation by variably acylated lipid A variants in different species (human, mice, hamster, horse) is still not a fully explained phenomenon, even though the solved co-crystal structures, mutagenesis studies, and molecular dynamics simulation analyses have provided profound insight into the molecular mechanisms of the species-specific action of lipid IVa on h- and mTLR4/MD-2. Lipid IVa, a tetra-acylated biosynthetic precursor of *E. coli* lipid A, can activate mTLR4/MD-2 by binding to the murine receptor complex in a manner similar to hexa-acylated *E. coli* lipid A, i.e., by exposing a large portion of its 2-*N*-linked β-hydroxyacyl chain on the surface of mMD-2 which shifts the Phe126 residue inwards and enables hydrophobic interactions with the second TLR4* ectodomain leading to heterodimerization [17]. Significant disparities in the binding mode of lipid IVa to hMD-2 (PDB code: 2E59), where the diglucosamine backbone is rotated by 180° and all acyl chains are fully buried in the binding groove, result in the conformational rearrangement in MD-2 and the relocation of Phe126 outwards which brings MD-2 into an “open” conformation unable to undergo heterodimerization [42]. This mode of binding is akin to that of the synthetic TLR4 antagonist Eritoran (PDB code: 2Z65) (Figure 4C,D). Hence, the acylation pattern of lipid A alone does not provide a sufficient basis for reliable and predictable structure–activity relationships in terms of species-specific recognition.

**Figure 5:**
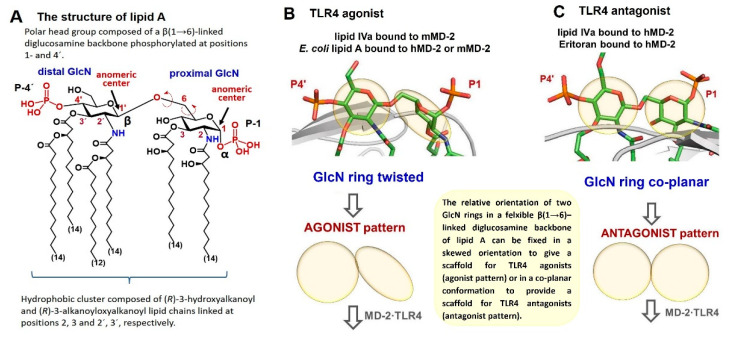
The chemical structure of *E. coli* lipid A and the 3D-tertiary structure of MD-2-bound lipid A in relation to biological activity. (**A**) Chemical structure of *E. coli* lipid A; (**B**) tertiary structure of the diglucosamine backbone of TLR4 agonist lipid A variants bound to MD-2/TLR4 complex; (**C**) tertiary structure of the diglucosamine backbone of TLR4 antagonist lipid A variants bound to MD-2; crystal-structure-based design of synthetic TLR4 ligands.

Biophysical studies have confirmed that the long-chain (*R*)-3-alkanoyloxyalkynoyl residues of lipid A stick snugly together to comprise a voluminous hydrophobic cluster, whereas the β(1→6)-linked diglucosamine skeleton of lipid A possesses a substantial degree of flexibility owing to the possibility of unobstructed rotation around β(1→6) glycosidic and exocyclic oxymethyl (-CH_2_O-) linkages (Figure 5A) [153,154]. Thus, protein binding – induced amendment of dihedrals (or torsion angles) around the three-bond β(1→6) glycosidic linkage in the common βGlcN(1→6)GlcN backbone of endotoxic hexa-acylated lipid A results in a repositioning of the “proximal” GlcN ring into a skewed relative orientation (Figure 5B) that might facilitate the exposure of a notorious 2*N*-acyl lipid chain on the surface of MD-2, which, in turn, prompts the dimerization of two ternary TLR4/MD-2/LPS complexes and the following initiation of the intracellular signaling cascade (Figure 4A,B) [155,156]. According to co-crystal structures (PDB: 3FXI/3VQ2/3VQ1), the two GlcN rings of the disaccharide backbone of a protein-bound agonist lipid A (*E. coli* lipid A bound to h- or mTLR4/MD-2 and lipid IVa bound to mTLR4/MD-2) adopt a skewed relative orientation (Figure 5B), whereas the two GlcN rings of an antagonist lipid A (PDB: 2E59, 2Z65) [42,152] are found to accept a co-planar arrangement (Figure 5C) [155].

We assumed that the poor predictability of biological activity of variably acylated lipid A variants (the prediction is commonly based on the number, length, and distribution pattern of lipid chains) can be bypassed by manipulating the inherent flexibility of the carbohydrate backbone of lipid A, which we deemed responsible for the species-specific activity at TLR4. To create versatile TLR4-modulating glycolipids able to either efficiently inhibit the TLR4-driven inflammation through binding to MD-2 or to potently induce the TLR4/MD-2-mediated signaling, both in a species-independent fashion, we designed novel TLR4 ligands based on the disaccharide scaffolds with a fixed rigidified molecular shape corresponding either to an agonist (pyranose rings in a skewed arrangement) [157,158] or antagonist pattern (pyranose rings in a co-planar arrangement) [155,159] (Figure 5). Worth mentioning is that all known TLR4 ligands developed so far had either an ordinary β(1→6) diglucosamine backbone or more bendable skeletons where one or both GlcN rings were exchanged with linear aglycons [160,161,162]. Moreover, a TLR4 agonist structurally unsimilar to lipid A was developed; however, this small molecule was exclusively mouse-TLR4-specific [163].

**Figure 6:**
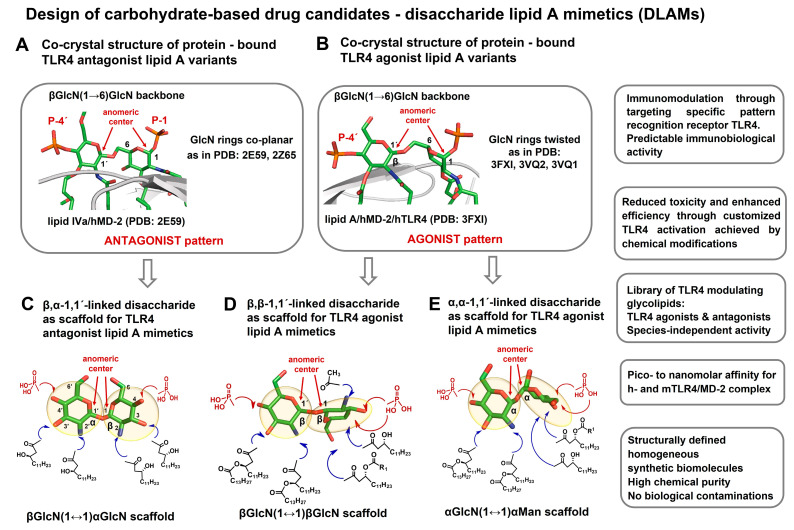
Co-crystal-structure-based design of disaccharide lipid A mimetics (DLAMs). (**A**) The co-crystal structures of hMD-2-bound lipid IVa and Eritoran (PDB codes: 2E59 and 2Z65, respectively) disclose a co-planar relative orientation of two GlcN rings comprising the β(1→6)-linked diglucosamine backbone of lipid A. This arrangement is achieved through rotation about flexible β(1→6) linkage. A specific co-planar relative orientation of the GlcN rings in a βGlcN(1→6)GlcN backbone in a protein-bound lipid A is designated an “antagonist pattern”. (**B**) The co-crystal structures of hMD-2/TLR4-bound *E. coli* lipid A (PDB code: 3FXI), mMD-2/TLR4-bound *E. coli* lipid A (PDB code: 3VQ2), and mMD-2/TLR4-bound lipid IVa (PDB code: 3VQ1) reveal a skewed relative orientation of two GlcN rings in the β(1→6)-linked diglucosamine backbone of protein-bound lipid A. This relative orientation of GlcN rings achieved through rotation about β(1→6) linkage is designated an “agonist pattern”. (**C**) Design of TLR4 antagonist lipid A mimetics. The co-planar molecular shape of β,α-1,1´-linked diglucosamine mirrors the 3D-topology of the βGlcN(1→6)GlcN backbone of hMD-2-bound TLR4 antagonists shown in (**A**). The sites of attachment of the lipid chains and phosphate groups for the synthesis of lipid A mimetics are represented by blue and red arrows, respectively. (**D**) Design of TLR4 agonist lipid A mimetics based on a β,β-1,1´-diglucosamine scaffold. The 3D-structure of a synthetic β,β-(1↔1´)-linked diglucosamine scaffold with two GlcN rings in a twisted relative orientation. The sites of possible chemical attachment of functional groups are indicated by arrows: the acylation/phosphorylation pattern of the distal GlcN ring corresponds to that of *E. coli* LPS, whereas the proximal GlcN ring has a variable acylation and phosphorylation pattern. (**E**) Design of TLR4 agonist lipid A mimetics based on an α,α -1,1´-linked disaccharide scaffold. The skewed molecular shape of an α,α-1,1´-linked diglucosamine mirrors the 3D-topology of a βGlcN(1→6)GlcN backbone of MD-2-bound TLR4 agonists shown in (**B**). The acylation and phosphorylation pattern of the distal GlcN ring corresponds to that of *E. coli* lipid A, whereas the proximal pyranose ring (Man) entails variable acylation and phosphorylation pattern. The sites of possible attachment of lipid chains are shown by blue arrows; the sites proposed for chemical phosphorylation are shown by red arrows. Images were generated with PyMol.

As stated above, the rotation about the β(1→6) glycosidic linkage (which is a distinctive feature of the βGlcN(1→6)GlcN backbone of lipid A) brings the proximal GlcN ring in a skewed orientation seen in all agonistic TLR4 co-crystal structures, i.e., for hexa-acylated *E. coli* lipid A bound to hMD-2/TLR4 (PDB code: 3FXI) and mMD-2/TLR4 (PDB code: 3VQ2), as well as for tetra-acylated lipid IVa in a complex with mTLR4 (PDB code: 3VQ1) (Figure 5B). In our approach, the flexible GlcNβ(1→6)GlcN backbone of natural lipid A was exchanged for a rigid nonreducing disaccharide scaffold imitating the 3D-molecular shape of the diglucosamine skeleton of a protein-bound lipid A, which is strikingly different for TLR4 agonist and antagonist ligands (Figure 6A,B). Along these lines, the β,α-1,1´-linked diglucosamine disaccharide with co-planar-arranged sugar rings mimicking the molecular shape of the diglucosamine backbone of hMD-2-bound antagonists lipid IVa and Eritoran (PDB codes: 2E59, 2Z65, respectively) was considered an excellent carbohydrate scaffold for synthetic TLR4 antagonists [155,159] (Figure 6A,C), whereas the α,α-1,1´-linked or β,β-1,1´-linked nonreducing disaccharide scaffolds reflecting the 3D-tertiary structure of the diglucosamine backbone of endotoxic lipid A found in the active [TLR4/MD-2/(*E.coli* lipid A)]_2_ complexes (PDB codes: 3FXI, 3VQ1, 3VQ2) (Figure 6B) showed a skewed relative orientation of two GlcN rings and thus were believed to provide a perfect scaffold for carbohydrate-based TLR4 agonists [157,164] (Figure 6D,E).

Accordingly, we performed the synthesis of a library of TLR4-modulating glycolipids, disaccharide lipid A mimetics (DLAMs), by replacing the flexible three-bond β(1→6)-diglucosamine frame of lipid A with a conformationally rigid two-bond (1↔1´)-linked disaccharide imitating the molecular shape of TLR4/MD-2-bound lipid A variants (co-planar arrangement of two sugar rings for TLR4 antagonists and twisted relative orientation of two sugar rings for TLR4 agonists) (Figure 6). The compounds are abbreviated DLAMs for “Disaccharide Lipid A Mimetics” with a prefix indicating the anomeric configuration of the 1,1´-glycosidic linkage (βα-DLAMs, αα-DLAMs, and ββ-DLAMs); the latter is decisive for the molecular shape of the scaffold.

## 7. TLR4 Antagonists Based on a βGlcN(1↔1)αGlcN Scaffold: Nanomolar Potency and Species-Independent Activity of βα-DLAMs

**Figure 7:**
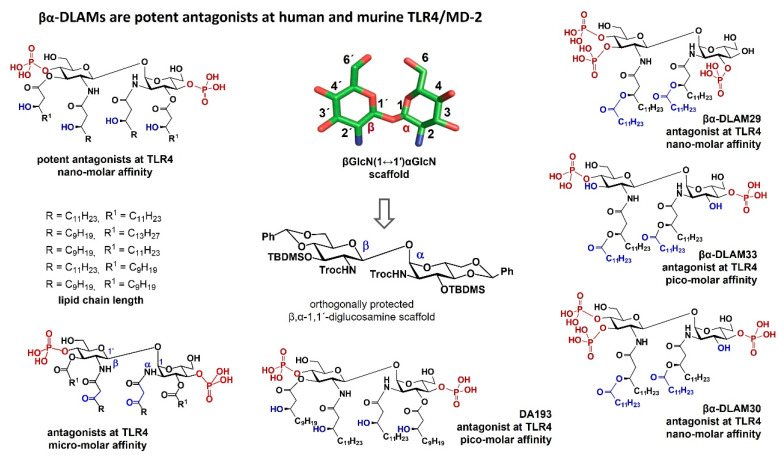
Synthetic disaccharide lipid A mimetics derived from βGlcN(1↔1)αGlcN disaccharide (βα-DLAMs).

Chemically synthesized, fully orthogonally protected, 1,1´-linked disaccharides were used as the starting point for the assembly of variably acylated and phosphorylated DLAMs. For the synthesis of TLR4 antagonists based on a β,α-1,1´-diglucosamine scaffold, the 2,2´-*N*-Troc, 3,3´-*O*-TBDMS, 4,6-, 4´,6´-*O*-benzylidene acetal-protected disaccharide was assembled by chemical glycosylation (Figure 7) [155]. One phosphate group was attached to each sugar unit, and the acylation pattern followed that of lipid IVa: each GlcN ring was 2-*N*-, 3-*O*-acylated by two long-chain fatty acids of different chemical structures and lengths (tetra-acylated compounds). The βGlcN(1↔1)αGlcN-based DLAMs were designed to match the geometry of the binding pocket of MD-2 (all lipid chains are fitted into the binding cleft of MD-2 analogous to lipid IVa and Eritoran in a complex with hMD-2).

In contrast to lipid IVa that acts as an antagonist at hTLR4 but as an agonist at mTLR4, the tetra-acylated βα-DLAMs could not induce the pro-inflammatory signaling in mouse cells owing to the co-planar shape and rigidity of their artificial disaccharide backbone [155,159]. Conversely, all conformationally confined βα-DLAMs performed as potent antagonists at both human and murine TLR4 [165,166]. Indeed, according to molecular dynamics simulations, the tetra-acylated TLR4 antagonist βα-DLAM DA193 was estimated to bind to the co-receptor protein hMD-2 ca. 20-fold more effectively than TLR4 agonist *E. coli* lipid A and 3-fold more strongly than TLR4 antagonist lipid IVa. NMR conformational studies revealed that the distance between the two 4, 4′ phosphate groups in βα-DLAM DA193 roughly corresponds to the distance P1–P4′ found in the native protein-bound lipid IVa.

The NOESY NMR experiments (performed for three variably acylated βα-DLAMs) revealed synΦα/synΦβ geometry around the βα-1,1-glycosidic linkage, which was in line with the double exo-anomeric conformation at both glycosidic torsions [159]. The co-planar conformation was not influenced by the length of multiple lipid chains. In this way, it was proved that the relative orientation of two GlcN rings in the βGlcN(1↔1)αGlcN backbone of the βα-DLAMs has a co-planar arrangement and thus mirrors the 3D-molecular shape of the native β(1→6) diglucosamine backbone of hMD-2-bound antagonists found in the co-crystal structures.

Therefore, fixing the tertiary structure of the carbohydrate backbone of lipid A in an ‘antagonist’ conformation (co-planar-arranged pyranose rings) through the replacement of a flexible βGlcN(1→6)GlcN backbone of native lipid A with a conformationally constrained β,α(1↔1)-linked diglucosamine scaffold surmounted the species-specific activity and gave rise to synthetic h- and mTLR4-antagonistic glycolipids of nanomolar affinity [165]. However, we observed some lipid chain length-dependent differences in the inhibition of the TLR4-mediated cellular responses by the βα-DLAMs in human and mouse cells, i.e., lipid A mimetic equipped with two C_14_ and two C_12_ lipid chains (DA193) showed the best antagonist potential at hTLR4, whereas the shorter-chain counterparts (i.e., 2xC_12_; 2xC_10_ lipid chains, DA255) demonstrated a higher affinity for mTLR4 [165] (Figure 7).

To further improve the affinity for MD-2 and to reduce the lipid chain length-driven species specificity in the binding of differently lipidated βα-DLAMs, we turned our attention to the unique structure of the lipid chains of a natural TLR4 antagonist, *R. sphaeroides* lipid A, which has one β-ketoacyl lipid chain (C_14_) attached at the proximal GlcN ring of the lipid A backbone (Figure 3). The β-keto acids and the β-keto amides commonly exist in a thermodynamic equilibrium with the corresponding enol forms. Although the equilibrium is heavily shifted in favor of the keto form, this ratio could also depend on the nature of the solvent and the protein surrounding of the binding pocket of MD-2. To address a possible influence of keto–enol tautomerism of the β-ketoacyl lipid chains within lipid A molecules, we synthesized a series of βα-DLAMs equipped with two 2*N*-linked β-ketoacyl chains of variable length and investigated their TLR4-inhibiting potential (Figure 7) [166].

Since the binding affinity of DLAMs was supposed to depend both on the lipid composition and on the shape of the hydrophobic region of the molecule, we again modified the acylation pattern and synthesized a series of potent TLR4 antagonists based on a βGlcN(1↔1)αGlcN scaffold acylated at positions 2-*N*, 2´-*N* by the long-chain-branched (*R*)-3-alkanoyloxyalkanoyl fatty acids, whereas hydroxyl groups at positions 3 and 3´ were let unsubstituted. Although a typical lipid A molecule has up to two phosphate groups, artificial hyperphosphorylation through appending an extra phosphate at the free OH-3 could increase ionic attraction to the positively charged Lys/Arg side chains on the rim of the binding pocket of MD-2. For that reason, a variable phosphorylation pattern (two, three, and four phosphate groups differently distributed along positions 3 and 4 on both GlcN units) was also included in this study (Figure 7). The antagonist potential of bis-phosphorylated and tri-phosphorylated molecules could be substantially enhanced and fluctuated from pico- to nanomolar concentrations.

Notably, a well-known antisepsis drug candidate E5564 (Eritoran) failed to improve survival in phase III clinical trials, despite an excellent antagonistic activity in vitro and in vivo [48], on account of several immunobiological drawbacks [167,168] as well as owing to the inherent instability of the labile glycosidic phosphate group P-1 turning the compound into inactive dephosphorylated metabolite [169]. Moreover, a possible neutralization by HDL was suggested as a conceivable explanation for the moderate therapeutic efficiency [169]. Therefore, the structure of the disaccharide skeleton of βα-DLAMs, where an unstable glycosidic phosphate group P-1 of lipid A/Eritoran is replaced by the far more durable secondary phosphate P-4, offers an unambiguous advantage for the creation of hydrolytically stable TLR4 antagonists.

## 8. TLR4 Agonists Based on αGlcN(1↔1)αMan and βGlcN(1↔1)βGlcN Scaffolds: Tailored Modulation of TLR4-Mediated Pro-inflammatory Signaling by αα-DLAMs and ββ-DLAMs

To design potent carbohydrate-based TLR4 agonists, we replaced the flexible β(1→6) glycosidic and exocyclic oxymethyl linkages present in the native βGlcN(1→6)GlcN frame of lipid A with an exceptionally stiff, nonreducing glycosidic linkage of either α,α or β,β configuration (Figure 6D,E). The unique rigidity of the 1,1-glycosidic linkages in the α,α- and β,β-linked nonreducing disaccharides that were supported by the carbohydrate-specific electronic effects was decisive for the immunobiological properties of the DLAMs based thereon.

The skewed relative orientation of the two pyranose rings in α,α- and β,β-1,1´-linked disaccharides is governed by the values of the torsion angles about the (1↔1´) glycosidic linkage which depends on the anomeric configuration and is influenced primarily by anomeric and exo-anomeric effects [170,171]. It has also been shown that the nature of functional/protecting groups and the sites of their attachment has no or little influence on the values of dihedrals around double glycosidic linkages [172]. Specific conformation of the αα-linked disaccharides has been confirmed by molecular dynamics simulations [172,173], and a predominant gauche–gauche conformation for substituted αα-trehalose [174] and its αGlc(1↔1)αMan analogue [175] was corroborated by X-ray structural studies and conformational analysis.

Similarly, it has been demonstrated that the dihedrals about the β,β-(1↔1´) linkage conferring a skewed topology to the β,β-1,1´-linked disaccharides (Figure 6D) are energetically favored and conserved in differently substituted derivatives [176,177,178,179]. A skewed molecular shape as a result of the staggered arrangement of the two β,β-1,1´-linked pyranoses (the staggered low-energy orientation in the positive direction (ca. 50°) places the C-1 *gauche* at the O-5 of the opposite ring) [171] would impose specific spatial orientation to functional groups connected to the twistedly arranged sugar rings (Figure 6). This would allow a presentation of specific long-chain (*R*)-3-hydroxyacyl residues and phosphate groups attached at the proximal sugar ring facing the secondary dimerization interface onto the surface of MD-2 which should make an efficient crosslinking of the second TLR4* receptor complex possible.

In contrast to the natural βGlcN(1→6)GlcN backbone of lipid A, which can spontaneously adjust its 3D-molecular shape (i.e., the relative orientation of GlcN rings) upon interaction with the MD-2/TLR4 complex, the relative stiffness of the α,α-(1↔1′) and ββ-(1↔1′) linkages confers exceptional rigidity of the disaccharide frames of DLAMs based thereon. The skewed relative orientation of the two pyranose rings in α,α-1,1´- and ββ-1,1´-linked disaccharides resembles the 3D tertiary structure of the MD-2-bound β(1→6)-linked diglucosamine backbone of endotoxically active lipid A (Figure 6B). Thus, the α,α-1,1´- and ββ-1,1´-linked disaccharide backbones of DLAMs also retain their specific molecular shape when bound by the MD-2/TLR4 complex.

**Figure 8:**
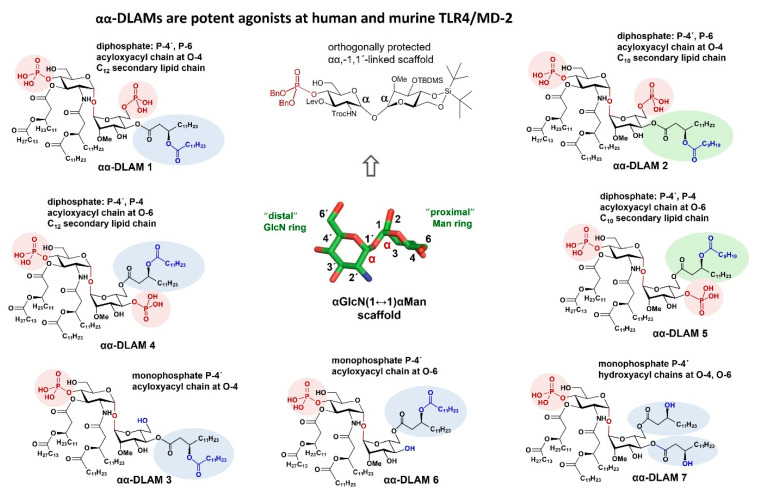
A library of synthetic disaccharide lipid A mimetics based on αGlcN(1↔1)αMan scaffold (αα-DLAMs).

**Figure 9:**
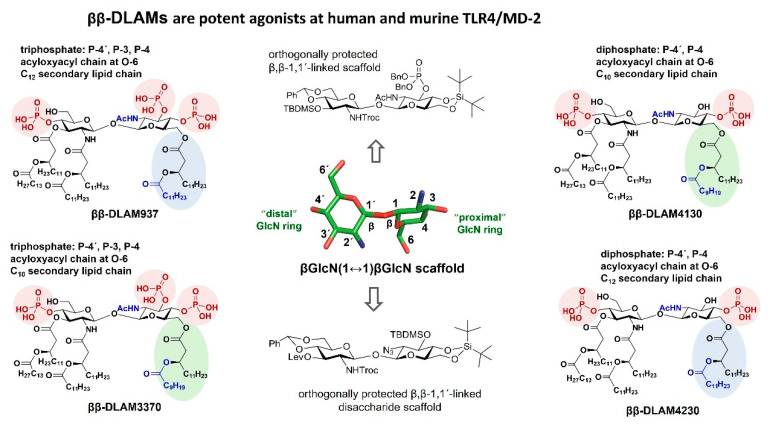
Potent disaccharide-based TLR4 agonists derived from a βGlcN(1↔1)βGlcN scaffold (ββ-DLAMs).

The sites of attachment of the phosphate groups and branched β-alkanoyloxyalkanoyl chains at the distal GlcN ring reflect the acylation and phosphorylation pattern of *E. coli* lipid A (acyloxyacyl residues at 2´-N and 3´-O, and a phosphate group at position 4´), whereas the proximal sugar moiety (Man for αα-DLAMs and GlcN for ββ-DLAMs) entails two β-hydroxyalkanoyl lipid chains and a variable number of phosphate groups that can be linked at positions 4 or 6 or 3/4 (Figure 8 and Figure 9). To estimate the relative contribution of ionic and hydrophobic interactions supplied by the phosphate groups and acyl chains, respectively, we synthesized four different groups of αα-DLAMs and two series of ββ-DLAMs.

Starting from a synthetically prepared, fully orthogonally protected αGlcN(1↔1)αMan disaccharide [156], three series of hexa-acylated, bisphosphorylated αα-DLAMs were synthesized (Figure 8). The compounds differ in the positioning of the phosphate group and acyl chains attached at the proximal mannose ring. Among diphosphate DLAMs where a second phosphate group is attached either at position 6 of the Man moiety (αα-DLAMs **1** and **2**) or at position 4 of the Man ring (αα-DLAMs **4** and **5**), the length of the secondary acyl chain in a (*R*)-3-acyloxyacyl residue linked to position 4 or 6 varies between C_12_ and C_10_ [157], respectively. The monophosphate αα-DLAMs **3**, **6,** and **7** lack a phosphate group at the Man moiety but differ in the sites of attachment of the branched β-acyloxyacyl chain. Accordingly, αα-DLAM **3** lipidated at position 4 is a monophosphate counterpart of αα-DLAM **1,** whereas αα-DLAM **6** with the acyloxyacyl chain at position 6 is a monophosphate counterpart of αα-DLAM **4** (Figure 8).

Upon interaction with the TLR4 complex, the four lipid chains linked at the distal GlcN ring were assumed to tightly bind to the hydrophobic binding pocket of MD-2, whereas the lipid chains and phosphate groups variably linked at the Man moiety in the αα-DLAM **1**–**7** were expected to face the secondary dimerization interface and to establish persistent ionic and hydrophobic interactions with the second TLR4* complex [156]. The alteration in the phosphorylation pattern (position 4 vs. position 6) at the Man moiety as well as variation in the acylation pattern (position 6 vs. position 4) and the length of the secondary acyl chains (C_12_ vs. C_10_) affected the efficiency and tightness of the TLR4 complex dimerization, which was subsequently translated in diverging downstream pro-inflammatory signaling [158].

The immunobiological properties of DLAMs were analyzed in a variety of cell types of human and mouse origin such as h- and mTLR4 transfected HEK293 cells, human mononuclear cells (MNC), dendritic cells (DCs), human acute monocytic leukemia cells (THP-1), cultured airway epithelial cell lines (Calu-3 and BEAS-2B), mouse macrophages, etc. The α,α-DLAMs and ββ-DLAMs could efficiently trigger the intracellular innate immune signaling that could be readily modulated by specific modification (i.e., the sites of attachment of the functional groups) at the proximal sugar ring of DLAMs. Picomolar concentrations of α,α- and ββ-DLAMs were already sufficient to induce the release of high levels of IL-6, IL-8, TNF-α, IL-1β, and MCP1 in the respective cell types. In contrast to native lipid A and lipid A analogues built on the flexible carbohydrate-containing or aglycon-based skeletons which often demonstrate species-specific activities, the TLR4-mediated pro-inflammatory signaling induced by DLAMs could be gradually and predictably modulated at both human and mouse TLR4 in a species-independent fashion.

The activation of the NF-κB-regulated signal transduction pathway by αα-DLAMs was analyzed over a broad concentration range through an assessment of the induction of secreted embryonic alkaline phosphatase (SEAP) in hTLR4/MD-2/CD-14-transfected HEK293 cells. The EC_50_ values for the αα-DLAM diphosphates **1, 2** and **4, 5** were in the picomolar range (70 to 400 pM), whereas the TLR4 activation potential of monophosphorylated αα-DLAMs **3, 6, 7** reflected their chemical structure lacking a phosphate group at the Man unit and dropped from a pico- to nanomolar concentration range (EC_50_ 3–60 nM) [158]. The decline in the TLR4-stimulating activity of the αα-DLAM monophosphates can be explained by the absence of a negatively charged phosphate group at the heterodimerization interface leading to feeble and inefficient dimerization [150] as well as by inferior recognition and transfer of molecules through CD14 [180].

The mTLR4 activation profile of αα-DLAMs in mTLR4/mMD-2-transfected HEK293 cells and in mouse macrophages (mBMDM) could be similarly regulated by altering the sites of acylation and phosphorylation at the proximal sugar ring, and the responsiveness of native murine immune cells to αα-DLAM challenge was again in the picomolar range for αα-DLAM diphosphates **1, 2** and **4, 5** (EC_50_ 90–320 pM for release of TNF-α) and, following the previously established structure–activity relationships, fell to nanomolar range in the case of αα-DLAM monophosphates **3, 6, 7** (EC_50_ 7–200 nM).

Picomolar doses of conformationally confined lipid A mimetics αα-DLAMs **1, 2** and **4, 5** also induced a concentration-dependent production of cytokines IL-6 and TNF-α in primary human immune cells (i.e., mononuclear cells (MNCs); EC_50_ 20–140 pM for release of IL-6 and 140–350 pM for release of TNF-α) [158]. The pro-inflammatory responses could be “programmed” and gradually modulated by adjusting the chemical structure of the carbohydrate-based molecules, i.e., by altering the length and position of acyl chains and the sites of attachment of phosphate groups at the proximal Man moiety. Along these lines, the relative potency of the structurally different series of αα-DLAMs to induce the release of pro-inflammatory cytokines in primary murine immune cells adequately correlated with the data obtained in human cells, thus providing proof for species-independent structure–activity relationships.

Our efforts in the simplification of the complex chemical structure of a native TLR4-stimulating ligand *Ra/Re*-LPS upon retaining full biological activity proved to be successful: the agonist DLAMs showed equal or higher potency in inducing the TLR4-specific signal transduction pathway as *Re*-LPS (Kdo_2_-lipid A) [158]. Although the composition of DLAMs is as minimal as that of lipid A (two pyranoses, up to six lipid chains, and one/two phosphate groups), the αα-DLAM diphosphates were 5- to 10-fold (depending on the chemical structure) more efficient in inducing the TLR4-mediated pro-inflammatory responses compared with *E. coli* lipid A and αα-DLAM monophosphates were more potent TLR4 activators compared with MPLA in both human and murine immune cells [158].

Further advanced modulation of TLR4-mediated pro-inflammatory responses in a variety of cell types was achieved with the synthesis of ββ-DLAMs—the TLR4-activating glycolipids based on a ββ-1,1´-linked diglucosamine scaffold differing in its 3D-topology from the 1,1´-linked αα- counterpart (Figure 9). Moreover, the primary chemical structure of the βGlcN(1↔1)βGlcN scaffold—with an equatorial 2-*N*-acetamide group instead of an axial 2-*O*-Me group in position 2 of the proximal sugar ring as well as a varying number of phosphate groups attached at positions 3/4 of the proximal GlcN residue—conferred specific immunobiological properties to the ββ-DLAMs based thereon [164]. As changes in the phosphorylation status of the carbohydrate backbone of native lipid A can markedly enhance or reduce the predisposition of LPS to interact with the TLR4 complex, we set out to investigate the influence of increased electronegativity of the sugar head group of DLAMs on the TLR4 activation and synthesized hyperphosphorylated ββ-DLAMs by attaching a third phosphate group at position 3 of the distal GlcN ring (along with 4, 4´ phosphate groups) (Figure 9). As expected, the characteristic geometry of the βGlcN(1↔1)βGlcN disaccharide conferred specific immunobiological behavior to the glycolipid immunomodulator ββ-DLAMs based thereon, and the TLR4-mediated cell activation induced by ββ-DLAMs could be fine-tuned by adding/removing a P-3 phosphate and/or shortening the length of a secondary acyl chain [164].

To summarize, the TLR4-mediated immunobiological activity of agonist DLAMs is governed by the tertiary structure of their conformationally confined 1,1´-linked disaccharide backbones and can be predictably modulated by facile chemical modifications, i.e., by adjustment of the length and distribution pattern of lipid chains on one sugar unit as well as the number and sites of attachment of phosphate groups. The TLR4 stimulatory potential of α,α- and ββ-DLAMs ranges from picomolar concentrations for multiply phosphorylated compounds to nanomolar concentrations for monophosphorylated DLAMs and surpasses the TLR4 activity of the most potent natural TLR4 agonist, *E. coli* lipid A. A further advantage of DLAMs is their improved hydrolytic stability achieved through the replacement of a labile glycosidic phosphate P-1 of lipid A for a stable secondary or primary phosphate group.

## 9. Conclusions

Given that PRRs including TLRs and their signaling pathways are highly conserved throughout evolution, the pharmacological exploitation of the TLR4 complex can be considered promising in the context of (i) chronic inflammatory disorders (atherosclerosis, arthritis, etc.); (ii) acute inflammation, i.e., SIRS and sepsis; (iii) airway inflammation of both chronic (asthma and allergy) and acute etiology (ALI); (iv) vaccine adjuvant development; and (v) oncological pathologies where both the activation and inhibition of TLR4 signaling could be of use as a stand-alone or adjuvant therapy.

Drawing on a crystal-structure-inspired design, we created a novel class of carbohydrate-based TLR4 agonists for tailored and controllable modulation of the TLR4-mediated signaling and disaccharide-derived TLR4 antagonists for inhibition of the TLR4-driven pro-inflammatory responses. The immunobiological activity of DLAMs is controlled by the configuration of the glycosidic linkage connecting the anomeric centers of constituting sugars which, in turn, determines the tertiary structure and the molecular shape of the nonreducing disaccharides applied as scaffolds for the assembly of glycophospholipid DLAMs. The relative spatial arrangement of two GlcN rings in the β,α-1,1´-linked diglucosamine was proved to be coplanar, which turned this disaccharide into a perfect scaffold for TLR4 antagonists, whereas α,α-1,1´- and β,β-1,1´-linked disaccharides with their skewed relative orientation of two sugar rings were exploited as scaffolds for TLR4 agonists. The 3D molecular shape of the disaccharide scaffolds (co-planar or tilted) served as the starting point for the design of lipid A mimetics, and the decision on the sites of attachment of the branched lipid chains and phosphate groups was made in agreement with the structural information provided by the co-crystal structures of the TLR4/MD-2/LPS (lipid A) complexes.

One of the major achievements of our investigation relates to surmounting the species specificity in ligand recognition by the TLR4 complex, which provides novel perspectives for a straightforward extrapolation of in vitro data obtained in human cellular models and human/murine immune cells to in vivo studies. To sum up, we generated a versatile library of fully synthetic disaccharide lipid A mimetics (DLAMs), embracing potent TLR4 antagonists with nanomolar affinity for MD-2 and powerful TLR4 agonists with picomolar affinity for TLR4/MD-2 as potential anti-inflammatory drug candidates for sepsis, acute and chronic inflammation as well as unique TLR4-activating immunomodulators with broad therapeutic potential for cancer and prophylactic capacity as vaccine adjuvants.

## Data Availability

Data sharing not applicable.

## Abbreviations

ALI	acute lung injury
BEAS-2B	human bronchial epithelial cell line
Calu-3	human lung adenocarcinoma cell line
CD14	cluster of differentiation 14
COPD	chronic obstructive pulmonary disease
DAMP	danger-associated molecular pattern
DCs	dendritic cells
DLAM	disaccharide lipid A mimetic
GPI	glycosyl phosphatidyl inositol
HEK293	human embryonic kidney 293 cells
HMGB1	high-mobility group box 1 protein
IFN-β	interferon-β
IL-1β	interleukin-1β
IRF3	interferon regulatory factor 3
Kdo	3-deoxy-D-*manno*-octulosonic acid
LBP	LPS-binding protein
LPS	lipopolysaccharide
LRR	leucine-rich repeat
MCP-1	monocyte chemoattractant protein-1
MD-2	myeloid differentiation factor 2
MNC	mononuclear cells
MPLA	monophosphoryl lipid A
MPL^®^	clinical-grade MPL^®^ adjuvant (GlaxoSmithKline)
MyD88	myeloid differentiation primary response 88
NF-ĸB	nuclear factor kappa B
PAMP	pathogen-associated molecular pattern
PDB	Protein Data Bank
PRR	pattern recognition receptor
SEAP	secreted embryonic alkaline phosphatase
SIRS	severe inflammatory response syndrome
THP-1	human acute monocytic leukemia cells
TIR	Toll-interleukin-1 receptor
TIRAP	TIR-domain-containing adaptor protein
TLR4	Toll-like receptor 4
TNF-α	tumor necrosis factor-α
TRAF	TNF-receptor-associated factor
TRAM	TRIF-related adaptor molecule
TRIF	TIR-domain-containing adapter-inducing interferon-β
